# Epidemiology of *Blastocystis* Infection: A Review of Data from Poland in Relation to Other Reports

**DOI:** 10.3390/pathogens12081050

**Published:** 2023-08-16

**Authors:** Monika Rudzińska, Katarzyna Sikorska

**Affiliations:** Department of Tropical Medicine and Epidemiology, Faculty of Health Sciences, Medical University of Gdańsk, 80-210 Gdańsk, Poland; ksikorska@gumed.edu.pl

**Keywords:** *Blastocystis*, prevalence, subtypes, human, animal, water, epidemiology, Poland

## Abstract

*Blastocystis* is a common gut protist of humans and various animals worldwide, with a high level of genetic diversity. Neither its zoonotic potential and transmission routes nor its pathogenicity are fully known. This fact, and the fact that *Blastocystis* is the most abundant eukaryote in human faeces, raises the question of its relevance to public health. Here, we summarise (in relation to other reports) the results of studies on the prevalence and genotypic variation of *Blastocystis*, which were carried out in animals, humans, and in water environments in Poland. In humans, the prevalence ranged between 0.14 and 23.6%, in some animals reached 58.97%, and in water environments was 5.1%. Seven subtypes were identified in humans (ST1-ST4, ST6, ST7, and ST9), of which ST3 was the most common. Among animals (wild, livestock, and pet animals), eleven STs were identified, with differential host specificity. Humans and animals shared ST1, ST2, ST3, ST6, and ST7, while ST1 and ST3 were present in humans, animals, and water sources. These observations indicate the possibility of *Blastocystis* transmission between animals and humans. Further studies should be continued in search of the sources and transmission routes of *Blastocystis* in order to prevent the spread of infections among humans and animals.

## 1. Background

*Blastocystis* spp. are anaerobic eukaryotic microorganisms that reside in the intestines of humans and many species of domestic and wild animals [1,2,3,4,5]. They are the most commonly detected eukaryotes in human faecal samples worldwide, especially in hot climates and countries/communities with low sanitary and hygienic standards [6]. Infection occurs (as in the case of enteric pathogens) by the faecal-oral route—in this case, by ingestion cysts that may be present in water or food or by direct contact with the reservoir of the microorganism. It is estimated that as many as 1 billion people worldwide are infected with *Blastocystis* [7].

These organisms exhibit a high degree of genetic variability—based on differences in the sequence of the gene encoding the small subunit of ribosomal RNA (SSU rRNA), 34 genetic groups called subtypes (ST) have been identified, although the validity of four of these STs (ST18–ST20, and ST22) is considered debatable. In humans, 14 subtypes (ST1–ST10, ST12, ST14, ST16, and ST23) have been reported, but four of them, ST1–ST4, account for more than 90% of human infections. ST5–ST8 are reported less frequently, and the other five are sporadic [7,8,9,10,11,12].

Epidemiological studies in non-human hosts have shown high infection rates in primates, pigs, domestic ruminants (including cattle, goats, and sheep), and birds [5,13,14]. Animals and humans who come into contact with them are often infected with the same subtypes, and some studies have shown identical sequences of *Blastocystis* isolated from animals and humans, raising the suspicion of a zoonotic nature of *Blastocystis* infections in humans [15,16,17,18,19,20,21,22]. However, reverse transmission, i.e., human to animal, has also been reported [23,24]. The impact of *Blastocystis* on human health ranges from that of a pathogen to a commensal or potentially beneficial organism. Usually, infection in humans is mild and self-limiting. Up to 50% of infected people may be asymptomatic carriers for months or years [25,26,27,28]. On the other hand, many people with *Blastocystis*-only invasion report symptoms, primarily gastrointestinal (mainly lack of appetite, nausea, loose stools, abdominal pain of varying severity, flatulence, bloating, and constipation), and less frequently, skin lesions or pruritus [29,30,31,32]. Additionally, a possible association of *Blastocystis* with the development of irritable bowel syndrome (IBS) and inflammatory bowel disease (IBD) has been reported [33,34,35,36].

The highly variable course of *Blastocystis* infection in humans, ranging from asymptomatic to severely symptomatic, may result from the fact that morphologically identical *Blastocystis* isolates are genetically very diverse (6–20% of the coding genes are unique to the subtype). This information has led to the suspicion that only certain subtypes have pathogenic potential. However, despite numerous studies, this has not been definitively confirmed, possibly due to intra-subtype variation, which may also influence the virulence or pathogenicity of the subtypes [37,38,39,40,41].

Moreover, an increasing number of studies on the human gut microbiome show both a higher richness and a higher evenness of the gut bacterial microbiota in people colonised with *Blastocystis*, which is beneficial for gut health. Hence, an emerging hypothesis is that *Blastocystis* colonisation of the intestines may be a sign of health rather than a disease, as previously thought [26,42,43,44].

Laboratory diagnosis of *Blastocystis* is a challenge. Molecular methods are the most accurate tool for detecting *Blastocystis*, but numerous countries still lack the proper facilities to perform them. Microscopy (routine coproscopic methods are usually helpful and valuable for diagnosing gastrointestinal parasites) in the case of *Blastocystis* may have reduced sensitivity due to the high morphological variability (at least four different morphological forms) and the instability of the microorganism’s cells. To increase the accuracy of the diagnosis, only experienced laboratory personnel should perform microscopy or a culture of *Blastocystis* should be performed in parallel [45,46].

Clinical diagnosis of blastocystosis is also challenging due to the lack of criteria distinguishing colonisation from disease. The indications for treatment are debatable, the more so that the treatment is often ineffective, with the eradication of the protozoa failing or the infection recurring [47,48,49,50].

The present work aimed to review the prevalence and distribution of *Blastocystis* subtypes in Poland’s human and non-human populations on the background of data from other countries.

## 2. Acquisition of Data

Articles on the prevalence and subtypes of *Blastocystis* identified in Poland were searched in the PubMed, Scopus, Web of Science, and Google Scholar databases using the following keywords: *Blastocystis*, prevalence, subtypes, human, animal, water, epidemiology, Poland.

All studies conducted in humans, non-human hosts, and environmental samples in which infection rates were determined and/or *Blastocystis* subtypes were identified (using coproscopic, culture, and/or molecular techniques) were considered. The search had no limitations regarding the start date and was conducted in January 2023. Our study contains some shortcomings. The main weakness of this review is its purely descriptive formula. This limitation results from the fact that there are not many published papers on *Blastocystis* in Poland. In addition, they concern heterogeneous, often selected study groups of small sample sizes and describe different ways of collecting and processing material. In Poland, standards for diagnosing *Blastocystis* infection have not yet been defined. There is neither obligation to include *Blastocystis* on the diagnostic list of parasitological reports nor to identify its subtypes. Usually, in medical laboratories, the presence of *Blastocystis* is determined based on a microscopic examination of stool samples, while in research and scientific laboratories culture and molecular methods are also used.

This limitation makes it challenging to set inclusion and exclusion criteria for the final review and perform a typical meta-analysis according to the adopted rules using the quality assessment scale for the selected articles. On the other hand, all included papers come from reliable research and health centres dealing with parasitic infections, including *Blastocystis*.

## 3. Results

The collected material shows that studies on the prevalence of *Blastocystis* in Poland were conducted mainly in humans, slightly less often in animals, and sporadically in samples from water environments. Table 1 and Table 2 display the infection rates and *Blastocystis* subtypes reported in humans, while Table 3 shows those reported in animals. Some analyses were carried out using microscopic methods, which do not allow for the identification of *Blastocystis* subtypes (Table 1). In contrast, other studies focused on determining *Blastocystis* subtypes, resulting in the lack of information on the prevalence of the microorganism in the study groups (Table 2).

Most of the studies were carried out in asymptomatic adult populations, and only a few studies also included groups of people with gastrointestinal symptoms, people travelling to hot climates, people dealing with animals, and children.

**Figure 1:**
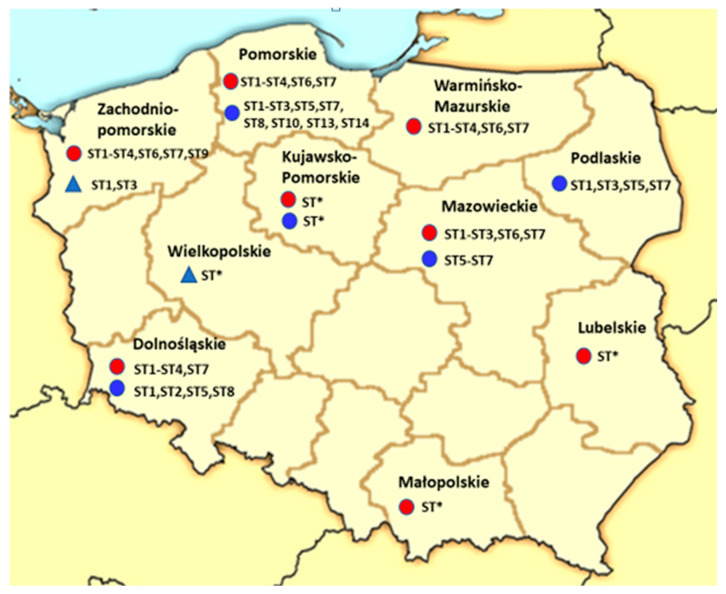
Regions of Poland where *Blastocystis* research was conducted [red dot—human research, blue dot—animal research, triangle—water environment; ST*—microscopy examination only, STs has not been identified; (details in Table 1, Table 2 and Table 3)].

Figure 1 shows a map of Poland with sampling sites and the *Blastocystis* subtypes identified in these regions in humans, animals, and water and water organisms.

## 4. The Prevalence of *Blastocystis* in Humans in Poland

The prevalence of parasitic infections in humans is related to the environmental, sanitary, and hygienic conditions in which they live. The specificity of parasitological research concerning organisms whose reservoir is the natural environment requires comparison with a possibly broad spectrum of data from the world. The result of the comparison may be the determination of universal factors affecting the level of infection with a given parasite species, as well as the identification of unique situations depending on the specificity of the environment, culture, and health in a given region of the world. This condition also applies to *Blastocystis* [76,77,78,79,80]. Numerous literature data indicate that the percentage of people infected with this microorganism in developed countries is usually lower (ranging from less than 1% to about 30%) than in developing countries, where it often exceeds 50% and may reach 70 or 80% [81,82]. Data from Poland, reporting infection rates of 0.14% to 23.6% in various study groups from 1955 to 2022 (Table 1), align with data from developed countries.

In Poland, the presence of *Blastocystis* in microscopic examinations of patients’ faeces was noticed as early as the 1970s in the laboratory of the Institute of Maritime and Tropical Medicine (IMTM) in Gdynia (now the Medical University of Gdańsk; MUG) Pomorskie Province. However, there was no infection registry until 1992, when the number of reports on the potential pathogenicity of *Blastocystis* began to increase. That year, the prevalence rate was found to be 1.34%, and then in the following years, this percentage increased almost continuously, reaching 18.8% in 2010. It should be emphasised that the frequency of infections with other gastrointestinal parasites (helminths and protozoa) among IMTM patients decreased during this period [54]. This 14-fold increase in infection cases can be partly explained by the specific group of IMTM patients, many of whom are people travelling (often repeatedly and for many months) to hot climate zones. The literature has often pointed out that staying in the tropics and subtropics is a risk factor for acquiring infections with gastrointestinal pathogens [83]. However, studies of stool samples from a broad population of children and adults with gastrointestinal symptoms and returnees from tropical and subtropical regions conducted in southern Poland (Małopolskie Province) between 2000 and 2006 did not find a single case of *Blastocystis* infection using similar microscopic techniques (direct saline and iodine staining smears) as in IMTM [84].

Similarly, no case of *Blastocystis* infection was recorded in Kujawsko-Pomorskie Province between 2000 and 2008, while in 2009–2014, the percentage of infected patients ranged between 0.14% and 0.87% [58]. On the other hand, among patients in Zachodnipomorskie Province studied between 1983 and 2012, *Blastocystis* was the second most frequent cause of intestinal parasite infections, with an average prevalence of only 0.89%. In this centre, sporadic cases of *Blastocystis* infection were recorded in 1988, the 1990s, and 2003–2008. However, a significant increase in infected patients (up to 4.8%) was recorded only in 2009–2012 [62].

Obtaining such different results may indicate a different level of ability to recognise *Blastocystis* in microscopic slides. However, it cannot be ruled out that *Blastocystis* may have been observed in microscopic slides but was not mentioned in the study reports, as this finding was considered clinically insignificant. The remarkable variation in the prevalence of *Blastocystis* in different communities within the same country has been reported by many authors; for example, 0.9% vs. 45.2% in Thailand [85,86], 18.5% vs. 40.7% in Malaysia [87,88], or 6.1% vs. 41.2% in France [77].

In Europe, similar rates of infection to those in Poland were reported in France (18.1%) and Spain (17.35%) [77,89] and in many countries outside Europe, such as Australia (19.1%) [90], Malaysia (18.5%) [87], Iran (14.5%) [39], Thailand (9.96%) [91], and the United States (7.19%) [92]. In Europe, higher values were observed only in Italy (34.13%; people with and without symptoms) [93] and in asymptomatic individuals in Cyprus (27.8%) [94], Belgium (30%) [95], and Ireland (56%) [42], which confirms that *Blastocystis* infections can be asymptomatic in many people. In the Czech Republic, among asymptomatic people who travelled outside Europe, *Blastocystis* infection was detected 10% more frequently than people who did not leave the country or travelled within Europe [96]. Similarly, in the Netherlands, among intercontinental travellers, the rate of *Blastocystis* infection upon return was 10% higher than before departure, and *Blastocystis* was one of the most common enteric organisms acquired during travel [77,83]. Many authors point out that the results of studies evaluating the frequency of *Blastocystis* infection in the studied population may be influenced by the methods used to detect the microorganism. Higher values were often reported when molecular methods, considered more sensitive and specific, were used [46,97]. This fact is confirmed by the studies mentioned above from Poland, in which microscopic methods resulted in an average infection rate of 5.8% (ranging from 0.14% to 18.8%) compared with 11.8% (ranging from 1% to 23.6%) for molecular methods (Table 1).

The available world literature shows that *Blastocystis* is commonly found in children, with the range of infection rate as wide as in adults, varying from 0.5% to more than 80%, depending on the populations studied (Table 4) and provided methods of examining the samples.

To the best of our knowledge, in Poland, the presence of *Blastocystis* was first described in 1955 in children (3–7 years old). The percentage of children who had *Blastocystis* detected by microscopic examination of a stool sample was 8%, and interestingly, in this paper, *Blastocystis* was still referred to as “a unicellular plant organism—non-pathogenic element” [51]. In another study of children (with symptoms of gastroenteritis) carried out in 1991, the percentage of infected children was 5%, while in the another study in 2011 (children without symptoms of parasitic diseases), it was higher and amounted to 10.7% [52,53]. In 2014, screening for *Blastocystis* in children from Warsaw kindergartens showed a prevalence of only 0.77% [57]. In each of the studies mentioned above, the children came from a different region in Poland. In European countries with sanitary and socio-economic conditions comparable to Poland (Slovakia, Switzerland, the Czech Republic, and France), *Blastocystis* was detected in children with a frequency ranging from 0.65% to 26.3% [77,99,100,103]. The analysis of the above reports shows that the higher prevalence values may have been influenced by the size of the group and the use of molecular methods in the study more than other factors related to the characteristics of the population. Many reports show that children are often asymptomatic carriers of *Blastocystis*, with a surprisingly high infection rate (Table 4). For example, an unprecedented 100% infection rate with this protistan was recorded in Senegalese children from villages where clean tap water is accessible for less than 20% of dwellings. In this study, over half of the subjects experienced gastrointestinal disorders [112].

## 5. *Blastocystis* Subtypes Identified in Humans in Poland

In Poland, seven out of fourteen *Blastocystis* subtypes (ST1–ST4, ST6, ST7, and ST9) recorded in humans were identified (Table 1 and Table 2). The vast majority of cases showed the presence of single subtypes, with only 4 cases showing mixed infections (ST1/ST3), and in 19 cases, the subtype could not be determined. The dominant subtype, identified as the most numerous in each of the studies conducted in Poland, was ST3, accounting for a little over 48% in total.

ST3 was the dominant subtype in people of both sexes, of different ages, both travelling outside Europe and never leaving Poland (Table 1 and Table 2). This finding aligns with results from various regions worldwide, where ST3 has been reported as the most common subtype [7,14,82,113]. Though in some countries, particularly in Asia (Iran, Pakistan, the Philippines, Thailand, and Turkey) as well as Libya and Nigeria, the dominance of ST1 has been reported [15,81,114,115,116], and in some papers from Europe (Spain, Denmark, and France), the most frequently detected subtype was ST4 [31,32,117]. The fact that ST3 is the dominant subtype in many countries, including highly urbanized regions where the risk of zoonotic transmission is reduced, supports the idea that ST3 is the most human-specific subtype and is primarily transmitted between people (due to human-to-human transmission) [118].

In Pomorskie and Zachodniopomorskie Provinces (north and north-western Poland) and Poland in general, the second most common subtype was ST2, accounting for slightly above 15% of cases, and the third was ST1, representing slightly above 13% of cases. However, in Warmińsko-Mazurskie and Mazowieckie Provinces (north-eastern and central Poland), the order of prevalence rates for these two subtypes was reversed, with ST1 being the second most common and ST2 being the third. A similar rule was also observed for ST3 and ST1 *Blastocystis* infection rates among the northern and central Italian populations [93]. Recent meta-analyses from Asia and the Americas have confirmed the overwhelming dominance of these two *Blastcystis* subtypes in humans. In Asia, ST3 is the most numerous and widely distributed subtype, being identified in 15 countries, with 10 having it as the most numerous. In the Americas, ST3 is the most numerous subtype, but ST1 is more widespread (reported in 10 out of 10 countries), followed by ST3 (in 9 out of 10 countries) [14,119].

Interestingly, among IMTM patients, ST2 was twice as common, and ST1 was found only in the group of people travelling outside Europe, mainly to Asia and Africa, and was not present in those who had never left Poland [66,68]. Different results were obtained in the group of subjects in Zachodniopomorskie Province, where none of those infected with ST1 travelled outside Poland [64]. Observations indicating that ST1 infections may be favoured by travelling to tropical and subtropical regions of the world were made in patients in the Netherlands [120], while no such correlation was found in asymptomatic volunteers studied in the Czech Republic [96].

ST4, ST6, and ST7 were identified much less frequently in all locations in Poland, accounting for 2%, 2.2%, and 2.47% of cases, respectively. ST9 was reported only once, and the remaining subtypes (sporadically reported in humans) were not detected in Poland (Table 1, Figure 1). ST4 is a subtype identified much more often in Europe, with a prevalence of almost 20%, compared to other regions where its proportion is 3–4% and in Africa less than 1% [81,82]. In Denmark, Spain, and France, ST4 was identified most often of all subtypes, accounting for 76%, 94.1%, and 63% of cases, respectively [31,32,117]. In Denmark and Spain, ST4 was the predominant subtype among symptomatic individuals with acute diarrhoea, while healthy individuals were not infected with this subtype [31,32]. Compared to the mentioned studies, in Poland, only 5 out of 11 studies (where subtypes were identified) reported the detection of ST4 (Table 1 and Table 2). Most infected patients from whom information was obtained (7 out of 8 people) did not leave Poland. Similarly, 7 out of 8 infected (not precisely the same group of people, just a coincidence of numbers) had gastrointestinal symptoms (diarrhoea and abdominal pain). The symptoms persisted for years in one of these patients, and this patient was never diagnosed or treated for blastocystosis [64,65,66].

ST6 and ST7 are less common subtypes in people around the world. Individually, most cases of ST6 were reported in Egypt, Nepal, Japan, and Malaysia, and the highest number of ST7 infections were reported in Nepal, Egypt, Japan, Malaysia, and Pakistan (from highest to lowest number of cases). On average, these subtypes were most frequently observed in Europe (ST6 at 1.7%, ST7 at 1.2%) and in South America (ST6 at 1.3%, ST7 at 1.1%) and less often in Asia (less than 1%) [8,82]. In Poland, ST6 infections in humans were reported in four studies and ST7 in eight studies, with both subtypes detected in single cases (in 1–4 people) (Table 1 and Table 2). These subtypes are more frequently found in animals, including livestock, and most commonly in wild and farmed birds; for this reason, they have been named avian subtypes [5]. It is assumed that their presence in other animals and humans is the result of acquiring the infection from birds. For instance, Greige described slaughterhouse workers infected with these subtypes due to contact with poultry faeces and tissues [21]. The fact that these subtypes occur at a similar frequency in humans in the highly diverse environments of Europe and South America suggests that identifying the sources that infect people with these subtypes requires further in-depth research. In Poland, the faeces of chickens, geese, ducks, and pigeons were examined for the presence of *Blastocystis* DNA (Table 3), and the detection of *Blastocystis* subtypes ST6 and ST7 was only recorded in chickens [70,71,74].

To the best of our knowledge, *Blastocystis* ST9 has so far been found in only seven people worldwide: a Danish patient who suffered from IBS [121]; three patients from Iran diagnosed with schizophrenia, including two patients with recurrent abdominal pain and diarrhoea and one without symptoms [122]; and three patients from Japan [123]. Notably, the *Blastocystis* sequences of ST9 from Iran were very similar to those of ST9 from Denmark, with a genetic divergence of 0.5% while 1.1–1.2% compared to those from Japan [122]. In Poland, ST9 was detected in a 60-year-old woman who did not experience gastrointestinal symptoms, resided in a large city, never drank unboiled water, or travelled outside Poland but had contact with dogs [64]. Comparing the ST9 sequences from different regions, the divergence between Japanese ST9 sequences and the sequence obtained in Poland was over 3%, while the divergence between Danish and Polish ST9 sequences was 1.8%. Unfortunately, the Polish ST9 sequence does not cover the same fragment as the Iranian one, so comparing them was impossible. Additionally, it is essential to note that the Polish and Iranian sequences are shorter than those obtained in Danish and Japanese studies. Therefore, the divergence between the sequences has different values than those reported by Sheikh et al. [122].

The reason that only four cases of mixed infection (ST3/ST1) were detected in Poland could be attributed to the methods used in the studies. Most of the studies relied on the barcoding method, which involves PCR amplification of the SSU-rDNA gene of *Blastocystis* with universal primers and subsequent sequencing [124]. This method does not allow or significantly impedes the detection of mixed *Blastocystis* infections [7]. Scanlan noted a high prevalence of ST1 in mixed infections and that ST1 was overlooked in these infections, possibly because ST2, ST3, and ST4 were preferentially amplified over ST1 [125]. Conversely, the detection of such mixed infections is possible (although only within nine subtypes, i.e., ST1–ST9) using polymerase chain reaction with ST-specific sequence-tagged-site primers (STS-PCR) [123]. In Poland, in the study of Rudzińska, which compared the effectiveness of both methods, all mixed infections were detected using STS primers. Direct sequencing of the barcode region allowed only one subtype to be detected: ST1 in a mixed sample of ST1/ST3, and ST5 in samples containing ST5/ST1 and ST5/ST3 [73]. Worldwide, a high frequency of mixed subtypes has been identified in Indonesia (47 cases) [126], the Philippines (42 cases) [127], and Italy (80 cases in immigrants from Africa, South America, and Asia) [93]. The most commonly reported mixed infections were ST3/ST1, and it is important to note that none of these studies used the barcoding method. Therefore, it might be that the frequency of mixed subtypes in other studies in Poland may be underestimated due to methodological issues. In Poland, all cases of mixed infections, which are believed to result from different sources of infection [93], have been found in people who travelled outside the European continent, to Asia and Africa. It has also been postulated that mixed infections may result from immunodeficiencies, which could facilitate co-colonization by more than one subtype [125]. However, data from Polish publications do not contain information on the immunological status of people with mixed infections.

## 6. *Blastocystis* Prevalence and Subtypes Identified in Non-Human Hosts in Poland

In Poland, research on non-human hosts covered various groups of animals, including farm animals (pigs and poultry), pet animals, zoo animals, and wild animals, including mammals, birds, and reptiles.

The prevalence of *Blastocystis* infection among non-human hosts was more variable than in humans (Table 3). The highest values were observed in non-human primates (NHPs) in the Gdańsk Zoo (58.97%) and in pigs (46.97%), whereas in some groups of animals (e.g., cats, rodents, wild ducks, and pigeons), *Blastocystis* was not found.

In total, 11 *Blastocystis* subtypes (ST1–ST3, ST5–ST8, ST10, ST13, and ST14) have been identified in non-human hosts in Poland, of which only ST13 has not been recorded in humans so far. Five subtypes have been detected in NHPs (ST1, ST2, ST3, ST5, ST8, and ST13), seven subtypes in Artiodactyla (ST1–ST3, ST5, ST7, ST10, and ST14), three in Suidae (ST1, ST3, and ST5), and also three in birds (ST5–ST7). In companion animals of Carnivora, *Blastocystis* ST7 was detected in one dog. In addition, sequences of *Blastocystis* that were not similar to any of the known subtypes of mammals (including humans) and birds (hence called non-mammal, non-avian subtypes; NMASTs) have been identified in reptiles (living in the zoo or kept at home) [128].

The results obtained in animals and human studies in Poland suggest the possibility of *Blastocystis* transmission between animals and humans, and, importantly, in both directions.

Some subtypes found in non-human hosts in Poland appear more host-specific, while others show lower host specificity. For example, ST13 was found only in monkeys; ST10 and ST14 only in sheep and goats; ST6 only in chickens. ST5 has been observed in a broader range of animal species, primarily in pigs, wild boar, and peccaries, as well as in 1 out of 7 wild wolves and 2 out of 90 wild bison, and in chickens. ST1 and ST3 were most prevalent in monkeys but were also occasionally detected in pigs, both wild and captive (from zoo) Artiodactyla. So far, in small ruminants used by humans, such as sheep, goats, and wild Artiodactyla, ST10 and ST14 were the most often reported subtypes, while ST1 and ST2 were found much less frequently [5]. In wild Artiodactyla living in Poland, ST1 (in red deer) and ST1, ST3, ST5, and ST7 (in European bison) were detected, i.e., different than those commonly reported in these animals (mainly ST10 and less frequently ST13 or ST14). According to our knowledge, so far, ST1 in deer has only been reported once in sika deer in China [129]. These wild ruminants, in which Polish researchers detected ST1 and ST3, came from the Białowieża National Park, where they live in the wild. However, during the winter, they have access to human-provided food such as hay and vegetables, and often forage on farmland that can be fertilised with manure containing livestock faeces. The diversity of food sources and environments these animals encounter during the winter months could lead to a higher likelihood of being exposed to different subtypes of *Blastocystis*. Perhaps this is the reason for the detection of ST1 and ST3 in the wild ruminants in Poland [75]. It is also worth mentioning that different subtypes of *Blastocystis* were found in Artiodactyla in Gdańsk Zoo (ST10, ST14) and in the Wrocław Zoo (ST1, ST2). However, cases of infection with different subtypes in animals of the same genus living in various regions/parts of the country have already been reported in the literature; for example, camels in Libya or goats in Malaysia [130,131]. This disparity may be the result of differences in animal management practices.

Similar differences were observed in NHPs; ST5 and ST8 were detected in the animals at Wrocław Zoo, while ST1, ST2, ST3, and ST13 were detected in the NHPs living at Gdańsk Zoo. So far, 13 *Blastocystis* subtypes (ST1–ST5, ST7–ST11, ST13, ST15, and ST19) have been identified in NHPs, with the high prevalence of ST1, ST2, and ST3. Interestingly in free-living NHPs, mostly ST1 and ST2 were reported (in Gdańsk Zoo, the most frequent was ST2, followed by ST3), while in captive NHPs, these and other subtypes were observed [5]. This fact is puzzling because it is in the wild, in natural conditions, where animals have greater opportunities for contact with each other (within species and between species) or with the environment contaminated with the faeces of other animals than in zoos, where they are isolated from each other in separate pens. Therefore, perhaps the greater diversity of *Blastocystis* subtypes in captive NHPs is due to the participation of people (animal keepers) who can transmit *Blastocystis* from other animals while caring for them.

In Gdańsk Zoo, the only subtypes present in both animals (NHPs) and humans (their caregivers) were ST1 and ST3. Moreover, an identical ST1 sequence isolated from three monkeys was found in one of the people who had contact with them, which provides strong evidence that transmission of *Blastocystis* has taken place between these monkeys and their caregiver, although it is impossible to determine its direction [63].

A similar phenomenon was noted at a UK zoo, where a high frequency of ST8 was observed in monkeys and their keepers. ST8 is extremely rarely reported in humans, hence the strong suspicion, almost certainty, that the caretakers of the monkeys contracted ST8 from the animals [16]. In another study on two groups of water voles, one completely free-living and the other temporarily brought into captivity by humans, it was found that wild water voles harboured ST4 only, whereas those who had periodic contact with humans also harboured ST1 [132].

Worldwide, ST5 is most frequently isolated from pigs and wild boar [22,133,134,135] and, to a lesser extent, from other livestock animals and birds, which is believed to be related to animal-to-animal transmission [130,131,136]. Similarly, in Poland, ST5 was isolated primarily from representatives of the Suidae family (pigs and wild boars) and collared peccaries [63,73]. In wild boars and peccaries, ST5 was the only subtype found, and in pigs, it was dominant (92.85%), confirming that the Suidae family is most likely a natural host for this subtype. In Poland, in addition to ST5, single ST3 and ST1 infections and mixed infections with these subtypes, i.e., ST5/ST3, ST5/ST1, and ST3/ST1, were found in pigs. Similarly, worldwide, ST3 and ST1 have been reported much less frequently in pigs [133]. This lower frequency raises the suspicion of possible transmission of these subtypes from humans to pigs, especially since in the Polish study, in the youngest animals, i.e., piglets up to 4 weeks of age, only ST5 was present [73]. The possibility of transmission of *Blastocystis* between pigs and humans is also indicated by a study of intensively reared pigs and their contacts in Australia. In these pigs, as in the Polish study, the dominance of ST5 and single cases of infection with ST3, ST1, and mixed infections with these subtypes were observed. Notably, apart from dominant ST3 and ST1, single ST5 infections were also found in animal keepers. Both animals and humans showed a high frequency of infection (76.7% and 83.3%, respectively) [20]. In Poland, no case of ST5 infection in humans has been reported so far, but the population of people dealing with pigs has not been studied.

It is suspected that the transmission of *Blastocystis* may also occur between humans and companion animals due to the reduction of the distance in contact between humans and their pets [19]. In Poland, a single case was reported where a dog and its owner with diarrhoea were infected with the same rare subtype (ST7) [69], which was previously described as potentially pathogenic [137]. Other dogs, cats, and some other species of small pet animals tested in Poland were not infected with *Blastocystis*. The lack of infection may be because all these animals had owners and were well cared for in terms of nutrition and veterinary care. Literature data show that the prevalence of *Blastocystis* in dogs and cats varies widely, and environmental factors such as ownership, homelessness, or staying in a shelter may affect the level of infection [65].

Although the research showed that pets in Poland did not harbour *Blastocystis*, exceptions were found in two bearded dragons and one leopard gecko. However, the *Blastocystis* sequences isolated from them do not correspond to any subtypes reported so far in humans, other mammals, or birds [65]. This situation was also noted in the case of *Blastocystis* sequences isolated from turtles from Gdańsk Zoo [63] and identified in reptiles by other researchers [1,138]. This difference in *Blastocystis* harboured by reptiles allows us to hope that they do not pose a threat to mammals (including humans) and birds; however, to confirm this, further research on reptiles and other animals and people in contact with them is necessary.

## 7. *Blastocystis* in the Water Environment in Poland

We have found only two reports from Poland regarding testing the water environment for the presence of *Blastocystis*. The first study focused on surface water samples from 46 sites of 36 water bodies in north-western Poland, including lakes, rivers, the Szczecin Lagoon (where river and sea water mixes), and the Baltic Sea. Water samples were collected four times a year. ST1 was detected only in lagoon water during the winter, while ST3 in two rivers and two lakes during the winter, spring and summer seasons. Interestingly, identical sequences of ST1 derived from human faeces and ST3 derived from human, pig, and cattle faeces were found in the GenBank database [139].

The second study did not directly concern water samples, but duck mussels (*Anodonta anatina*) and swollen river mussels (*Unio tumidus*) originated from an urban reservoir lake in central-west Poland. It is a fairly shallow reservoir, with an average depth of 3 m and a maximum depth not exceeding 5 m. The water residence time in the reservoir is approximately 34 days, and it collects water from nearby rivers that may be polluted with sewage discharge. In this study, *Blastocystis* was not detected in duck mussels, while it was found in 5.1% of the samples of swollen river mussels. The tests in this study were performed using microscopic methods, such as direct wet and hematoxylin–eosin stained smears, but subtyping was not conducted [140].

Worldwide, the presence of *Blastocystis* DNA in the aquatic environment, including drinking and surface water, has been reported multiple times in different locations. The subtypes commonly identified in humans (ST1–ST4) were most frequently detected, while the subtypes rarely identified in humans (ST7, ST8, and ST10) were found much less frequently [5,6,141,142,143,144,145]. Many of these reports strongly indicate waterborne *Blastocystis* infections in humans. For example, a study of military personnel in Thailand found that 44.1% of people in a military unit that used untreated water were infected, and the risk of infection from consuming this water was three times higher than those using other water sources [146]. Another support could be the detection of the same subtypes (ST1 and/or ST4) in various types of water (water from the water tank at school, tap water, and river water) and in people and animals using it [6,142,143,147]. In addition, the presence of viable cysts in the water discharged from a sewage treatment plant [148,149], as well as the detection of *Blastocystis* DNA in seafood, marine fish, and marine mammals, indicate the potential for multi-directional transmission of *Blastocystis* between humans and animals via water [4,150]. However, to conclusively confirm such waterborne transmission of *Blastocystis*, obtaining the same sequences of subtypes isolated from water sources and individuals using that water would be essential.

The results of both Polish studies indicate highly probable contamination of the tested waters with faeces (from both human and/or non-human hosts) and a potential risk of acquiring infection by people during recreational water activities (in general, water from rivers and lakes that has not been treated or boiled is not used for human consumption in Poland). The examined mussel species, in which *Blastocystis* was detected in Poland, feed by filtering the water (they can filter over 2 L of water per hour per shellfish) and thus act as a natural concentration system [140]. The detection of *Blastocystis* in their digestive tracts is, therefore, evidence of the presence of this microorganism in the water where the mussels lived. Previously, *Blastocystis* was detected in bivalve molluscs of the genus *Donax* from the Peruvian northern coast [150]. These above-mentioned reports from Poland, which initiated the study of the water bodies for *Blastocystis* and confirmed the presence of this protist in the water environment, highlight the need to increase the investigation of various elements of the water-related environment. This direction of study is crucial to assess the potential risk of acquiring *Blastocystis* infection by people and animals via water.

The authors intended to present, for the first time, a summary of the epidemiological situation of *Blastocystis* in Poland, based on published research results. Coincidentally, this study highlighted some weaknesses of the studies conducted so far. However, due to the unresolved problem of the disease potential of *Blastocystis*, unclear mode of transmission, widespread international travel, and migration pressure, this work (despite the above-mentioned shortcomings) seems to be an essential contribution to expanding our knowledge of possible interactions among these factors in the aspect of the health safety of humans, animals, and the environment.

## 8. Summary and Perspectives

Our study summarizes the epidemiological knowledge about the prevalence and distribution of *Blastocystis* subtypes in Poland, as well as the sources and routes of its transmission.

The collected data show that the frequency of *Blastocystis* infection in humans in Poland is similar to that observed in other developed countries, with ST3 being the most common subtype in humans.

The observed low host specificity of ST1 and ST3, and especially the finding of identical ST1 sequences in zoo monkeys and their caregiver, indicates that transmission of *Blastocystis* between humans and monkeys is highly possible.

The high prevalence of *Blastocystis* in pigs, wild boars, and peccaries, with the overwhelming dominance of ST5, confirms that Suidae are likely the natural hosts of ST5. The presence of single ST3 and ST1 infections, as well as mixed infections with these two subtypes, suggests the probable anthroponotic direction of the infection in pigs. However, further in-depth studies of both hosts are needed to fully understand the issue of human–pig transmission of this protist.

The absence of *Blastocystis* in dogs (with one exception) and cats suggests that these pet animals are not natural hosts of the microorganism and do not constitute a significant reservoir of *Blastocystis* infection to their owners or keepers. Similarly, findings in reptiles *Blastocystis* genotypes that differ from those found in humans, other mammals, and birds argue that reptiles do not currently pose a reservoir of infection for humans.

The presence of *Blastocystis* in surface waters and mussels indicates that interactions between humans and animals, including via water, can risk spreading *Blastocystis* infection among users of polluted waters.

Furthermore, the observation that travelling to tropical regions may favour infection with some subtypes of *Blastocystis* (especially ST1) is important in the epidemiological and clinical aspects, especially in the era of dynamic growth in tourism after the downturn caused by the COVID pandemic.

To gain a comprehensive understanding of the sources of infection and transmission routes of *Blastocystis*, further well-designed, in-depth studies are necessary. Additionally, the essential direction in future studies on *Blastocystis* human infections should probably concern lateral gene transfers (LGT) among *Blastocystis* [151]. The LGT could shed light on the relation between transferred genes encoding secreted proteins potentially involved in adapting *Blastocystis* to the gut environment. These proteins can affect the gut microbiome and are involved in the inflammation state of the gut. This genetic dependence can affect the condition of patients and, in effect, act as a factor differentiating the number of cases of symptomatic blastocystosis noted in different countries/regions.

## Figures and Tables

**Table 1:** *Blastocystis* infection frequency and subtypes identified in patients from different groups and regions in Poland.

Study Group; Study Time (If Given ^A^)	No. of Subjects (*n*)	Method (s)	No. of Positive Subjects		Identified STs (*n*; If Applicable)	Region of Poland (Main Town)	Reference
			*n*	%			
Kindergarten children 3–7 years old 1953–1954	1504	microscopy	not given	8	na	Lubelskie (Lublin)	[51]
Children with the symptoms of gastroenteritis 1986–1988	257	microscopy	13	5	na	Mazowieckie (Warsaw)	[52]
Children without symptoms of parasitic diseases 2010	998	microscopy	108	10.7	na	Warmińsko-Mazurskie (Olsztyn)	[53]
Symptomatic/asymptomatic patients of IMTM 1992–2010	19,261	microscopy	2315	1.34–18.8 ^B^	na	Pomorskie (Gdańsk)	[54]
Soldiers returning from peacekeeping missions 2008–2010	913	microscopy	140	15.33	na	Zachodniopomorskie (Szczecin)	[55]
Volunteers	259	microscopy; barcoding	17 31	6.6 12	ST1 (9), ST2 (nng), ST3 (10), ST4 (nng), ST6 (1),ST7 (2), STun (3)	Warmińsko-Mazurskie (Olsztyn)	[56]
Kindergarten children 3–6 years old 2014	1823	microscopy	14	0.77	na	Mazowieckie (Warsaw)	[57]
Voivodeship Sanitary–Epidemiological Station 2000–2014	24,609	microscopy	27	0.14–0,87 ^C^	na	Kujawsko-Pomorskie (Bydgoszcz)	[58]
Immunocompetent (ic) and immunodeficient (id) patients 2007–2015	283; (46 ic, 237 id)	barcoding	3	1	ST3 (2 ic), ST2 (1 id)	Mazowieckie (Warsaw)	[59]
Symptomatic Asymptomatic Healthy persons	72 40 101	barcoding	17 4 22	23.6 10 21.7	ST1, ST2, ST3, ST4, ST7 ST1, ST2, ST3, ST7 ST1, ST2, ST3, ST7; (nng)	Dolnośląskie (Wrocław)	[60]
Young volunteers	384	barcoding	39	10	ST3, ST7, ST1, ST2 (in order of quantity, details not given)	Mazowieckie (Warsaw)	[61]
Patients of Pomeranian Medical University in Szczecin 1983–2012	9760	microscopy		0.3–1.19 ^D^	na	Zachodniopomorskie (Szczecin)	[62]
Zoo animal caregivers 2018–2019	35	barcoding	6	17.14	ST1 (3), ST3 (3)	Pomorskie (Gdańsk)	[63]
Pre- and perimenopausal women	425	barcoding	26	6.1	ST1 (5), ST2 (7), ST3 (7), ST4 (3), ST6 (2), ST7 (1), ST9 (1)	Zachodniopomorskie (Szczecin)	[64]
Pet animal owners 2018–2020	67	barcoding	3	4.5	ST3 (1), ST4 (1), ST7 (1)	Pomorskie (Gdańsk)	[65]

^A^ Some authors did not specify the time period in which they conducted the research, see publication date; ^B^ *Blastocystis* frequency range in years 1992–2010, ^C^ in years 2009–2014, ^D^ in years 1983–2012; na—not applicable, performed using microscopy only; nng—number not given; STun—subtype failed to be identified.

**Table 2:** *Blastocystis* subtypes identified in patients from Poland in studies conducted using molecular methods only on *Blastocystis*-positive individuals (order by year of publication).

Study Group; Study Time (If Given)	No. of Subjects (*n*)	Method	Identified STs (*n*)	Region of Poland (Main Town)	Reference
Symptomatic/asymptomatic patients of IMTM; 2005–2010	147	STS PCR	ST1 (16), ST2 (28), ST3 (84), ST4 (4), ST1/ST3 (2), STun (13)	Pomorskie (Gdańsk)	[66]
Patients of National Institute of Hygiene (PZH) and Hospital for Infectious Diseases *	25	barcoding	ST1 (3), ST2 (2), ST3 (15), ST6 (2), ST7 (1), STun (3)	Mazowieckie (Warsaw)	[67]
Symptomatic/asymptomatic patients of IMTM; 2012–2013	122	STS PCR	ST1 (16), ST2 (24), ST3 (72), ST6 (4), ST7 (4), ST1/ST3 (2)	Pomorskie (Gdańsk)	[68]
Two owners of a dog; 2017	2	barcoding	ST3 (1), ST7 (1)	Pomorskie (Gdańsk)	[69]

* Authors did not specify the period they conducted the research; see publication date; STun—subtype failed to be identified.

**Table 3:** *Blastocystis* infection frequency and subtypes identified in animals from various regions in Poland (order by year of publication); all studies performed using molecular methods.

Study Group; Study Time (If Given ^A^)	No. of Subjects (*n*)	No. of Positive		ST (*n*—If Given)	Region of Poland	Reference
		*n*	%			
Wild ducks	15	0	-	-	Mazowieckie	[70]
Pigeons	25	0	-	-		
Free-range chickens	41	11	26.8	ST6 (5), ST7 (6)		
Chickens 2016–2019	100	ng	ng	ST5, ST6, ST7	Mazowieckie	[71]
Primates and Artiodactyla from Wrocław ZOO	44 36	ng ng	ng ng	ST5, ST8 ST1, ST2 ^B^	Dolnośląskie	[72]
Pigs (15 farms) 2017–2018	149	70	46.97	ST1, ST5 ^B^, ST3/ST1, ST5/ST1, ST5/ST3, STun (numbers vary depending on the PCR method used)	Pomorskie	[73]
Domestic dogs 2017	31	1	3.2	ST7	Mazowieckie	[69]
Geese (five flocks) 2016–2019	989	74	7.48	ST has not been studied	Kujawsko-Pomorskie	[74]
Animals from Gdańsk ZOO 2018–2019	201	54	26.86	ST1 (4), ST2 (7), ST3 (6), ST5 (17), ST8 (1), ST10 (2), ST13 (6), ST14 (6), NMASTs (5)	Pomorskie	[63]
Wild Artiodactyla and Carnivora from Białowieża Primeval Forest 2018–2020	102 11	8 1	7.8 9	ST1 (3), ST3 (1), ST5 (3), ST7 (1) ST5 (1)	Podlaskie	[75]
Pet animals (dogs, cats, rodents, reptiles) 2018–2020	145	3 ^C^	2.1	NMAST (3)	Pomorskie	[65]

^A^ Some authors did not specify the period in which they conducted the research, see publication date; ^B^ dominant subtype; ^C^ only lizards were *Blastocystis*-positive; NMAST—not a mammal, not avian ST; STun—subtype failed to be identified; ng—not given.

**Table 4:** Frequency of *Blastocystis* infection in children from different countries.

No. of Subjects (*n*)	Infection Rate (%)	Country	Reference
372; 106	0.5–45.2	Thailand	[86,98]
2000	0.39–0.95	Slovakia	[99]
572	4.2	Switzerland	[100]
1760	10.6	Malaysia	[101]
9	11.1	North Cyprus	[94]
not given	11.9	Sri Lanka	[102]
52	15	Czechia	[103]
40	15	Jordan	[103]
265; 258	15.8; 39.22	Colombia	[10,104]
351	25.6	Angola	[105]
15	26.3	France	[77]
227; 123; 172	38.3; 40.7; 86.67	Brazil	[106,107,108]
303	37.9	Turkey	[109]
204	40.7	Philippines	[110]
51	45	Azerbaijan	[103]
59	47	Sudan	[103]
15	53	Tanzania	[103]
27; 199	55; 84	Nigeria	[103,111]
731; 93	51.7–100; 100	Senegal	[8,112]

## Data Availability

No new data were created or analysed in this study. All presented data are available in the cited publications.

## References

[1] Yosahikawa H., Koyama Y., Tsuchiya E., Takami K. (2016). *Blastocystis* phylogeny among various isolates from humans to insects. Parasitol. Int..

[2] Farah Haziqah M.T., Nur Asyiqin M.N., Mohd Khalid M.K.N., Suresh K., Rajamanikam A., Chandrawathani P., Mohd Zain S.N. (2017). Current status of *Blastocystis* in cockroaches. Trop. Biomed..

[3] Martínez-Barbabosa I., Gutiérrez-Cárdenas E.M., Hamdan-Partida A., Bustos-Martínez J., Shea M. (2018). Presence of *Blastocystis* spp. in the mollusc *Crassostrea virginica*, in Mexico City. Rev. De Salud Anim..

[4] Gantois N., Lamot A., Seesao Y., Creusy C., Li L.L., Monchy S., Benamrouz-Vanneste S., Karpouzopoulos J., Bourgain J.L., Rault C. (2020). First report on the prevalence and subtype distribution of *Blastocystis* sp. in edible marine fish and marine mammals: A large scale-study conducted in atlantic northeast and on the coasts of Northern France. Microorganisms.

[5] Hublin J.S.Y., Maloney J.G., Santin M. (2021). *Blastocystis* in domesticated and wild mammals and birds. Res. Vet. Sci..

[6] Eroglu F., Koltas I.S. (2010). Evaluation of the transmission mode of *B. hominis* by using PCR method. Parasitol. Res..

[7] Stensvold C.R., Clark C.G. (2016). Current status of *Blastocystis*: A personal view. Parasitol. Int..

[8] Khaled S., Gantois N., Ly A.T., Senghor S., Even G., Dautel E., Dejager R., Sawant M., Baydoun M., Benamrouz-Vanneste S. (2020). Prevalence and subtype distribution of *Blastocystis* sp. in Senegalese school children. Microorganisms.

[9] Maloney J.G., Santin M. (2021). Mind the gap: New full-length sequences of *Blastocystis* subtypes generated via oxford nanopore minion sequencing allow for comparisons between full-length and partial sequences of the small subunit of the ribosomal rna gene. Microorganisms.

[10] Osorio-Pulgarin M.I., Higuera A., Beltran-álzate J.C., Sánchez-Jiménez M., Ramírez J.D. (2021). Epidemiological and molecular characterization of *Blastocystis* infection in children attending daycare centers in Medellín, Colombia. Biology.

[11] Jinatham V., Maxamhud S., Popluechai S., Tsaousis A.D., Gentekaki E. (2021). *Blastocystis* One health approach in a rural community of Northern Thailand: Prevalence, subtypes and novel transmission routes. Front. Microbiol..

[12] Baek S., Maloney J.G., Molokin A., George N.S., Cortés Vecino J.A., Santin M. (2022). Diversity of *Blastocystis* subtypes in horses in Colombia and identification of two new subtypes. Microorganisms.

[13] Alfellani M.A., Jacob A.S., Perea N.O., Krecek R.C., Taner-Mulla D., Verweij J.J., Levecke B., Tannich E., Clark C.G., Stensvold C.R. (2013). Diversity and distribution of *Blastocystis* sp. subtypes in non-human primates. Parasitology.

[14] Nemati S., Zali M.R., Johnson P., Mirjalali H., Karanis P. (2021). Molecular prevalence and subtype distribution of *Blastocystis* sp. in Asia and in Australia. J. Water Health.

[15] Thathaisong U., Worapong J., Tan-ariya P., Viputtigul K., Mungthin M., Sudatis A., Noonai A., Leelayoova S. (2003). *Blastocystis* isolates from a pig and a horse are closely related to *Blastocystis hominis*. J. Clin. Microbiol..

[16] Stensvold C., Alfellani M., Nørskov-Lauritsen S., Prip K., Victory E., Maddox C., Nielsen H., Clark C. (2009). Subtype distribution of *Blastocystis* isolates from synanthropic and zoo animals and identification of a new subtype. Int. J. Parasitol..

[17] Yoshikawa H., Wu Z., Pandey K., Pandey B.D., Sherchand J.B., Yanagi T., Kanbara H. (2009). Molecular characterization of *Blastocystis* isolates from children and rhesus monkeys in Kathmandu, Nepal. Vet. Parasitol..

[18] Parkar U., Traub R.J., Vitali S., Elliot A., Levecke B., Robertson I., Geurden T., Steele J., Drake B., Thompson R.C.A. (2010). Molecular characterization of *Blastocystis* isolates from zoo animals and their animal-keepers. Vet. Parasitol..

[19] Nagel R., Cuttell L., Stensvold C.R., Mills P.C., Bielefeldt-Ohmann H., Traub R.J. (2012). *Blastocystis* subtypes in symptomatic and asymptomatic family members and pets and response to therapy. Intern. Med. J..

[20] Wang W., Owen H., Traub R.J., Cuttell L., Inpankaew T., Bielefeldt-Ohmann H. (2014). Molecular epidemiology of *Blastocystis* in pigs and their in-contact humans in Southeast Queensland, Australia, and Cambodia. Vet. Parasitol..

[21] Greige S., El Safadi D., Bécu N., Gantois N., Pereira B., Chabé M., Benamrouz-Vanneste S., Certad G., El Hage R., Chemaly M. (2018). Prevalence and subtype distribution of *Blastocystis* sp. isolates from poultry in Lebanon and evidence of zoonotic potential. Parasites Vectors.

[22] Pintong A., Sunyanusin S., Prasertbun R., Mahittikorn A., Mori H., Changbunjong T., Komalamisra C., Sukthana Y., Popruk S. (2018). *Blastocystis* subtype 5: Predominant subtype on pig farms, Thailand. Parasitol. Int..

[23] Pakandl M., Koudela B., Vitovec J. (1993). An experimental infection of conventional and gnotobiotic piglets with human and porcine strains of *Blastocystis*. Folia Parasitol..

[24] Iguchi A., Ebisu A., Nagata S., Saitou Y., Yoshikawa H., Iwatani S., Kimata I. (2007). Infectivity of different genotypes of human *Blastocystis hominis* isolates in chickens and rats. Parasitol. Int..

[25] Andersen L.O., Stensvold C.R. (2016). *Blastocystis* in health and disease: Are we moving from a clinical to a public health perspective?. J. Clin. Microbiol..

[26] Audebert C., Even G., Cian A., Loywick A., Merlin S., Viscogliosi E., Chabe M. (2016). Colonization with the enteric protozoa *Blastocystis* is associated with increased diversity of human gut bacterial microbiota. Sci. Rep..

[27] Beghini F., Pasolli E., Truong T.D., Putignani L., Cacciò S.M., Segata N. (2017). Large-scale comparative metagenomics of *Blastocystis*, a common member of the human gut microbiome. ISME J..

[28] Deng L., Wojciech L., Gascoigne N.R.J., Peng G., Tan K.S.W. (2021). New insights into the interactions between *Blastocystis*, the gut microbiota, and host immunity. PLoS Pathog..

[29] Bálint A., Dóczi I., Bereczki L., Gyulai R., Szucs M., Farkas K., Urbán E., Nagy F., Szepes Z., Wittmann T. (2014). Do not forget the stool examination!—Cutaneous and gastrointestinal manifestations of *Blastocystis* sp. infection. Parasitol. Res..

[30] Dagci H., Kurt Ö., Demirel M., Mandiracioglu A., Aydemir S., Saz U., Bart A., VAN Gool T. (2014). Epidemiological and diagnostic features of *Blastocystis* infection in symptomatic patients in Izmir province, Turkey. Iran. J. Parasitol..

[31] Domínguez-Márquez M.V., Guna R., Muñoz C., Gómez-Muñoz M.T., Borrás R. (2009). High prevalence of subtype 4 among isolates of *Blastocystis hominis* from symptomatic patients of a health district of Valencia (Spain). Parasitol. Res..

[32] Stensvold C.R., Christiansen D.B., Olsen K.E.P., Nielsen H.V. (2011). *Blastocystis* sp. subtype 4 is common in Danish Blastocystis-positive patients presenting with acute diarrhea. Am. J. Trop. Med. Hyg..

[33] Poirier P., Wawrzyniak I., Vivarès C.P., Delbac F., El Alaoui H. (2012). New insights into *Blastocystis* spp.: A potential link with irritable bowel syndrome. PLoS Pathog..

[34] Sekar U., Shanthi M. (2015). Recent insights into the genetic diversity, epidemiology and clinical relevance of *Blastocystis* species. J. Med. Res..

[35] Shirvani G., Fasihi-Harandi M., Raiesi O., Bazargan N., Zahedi M.J., Sharifi I., Kalantari-Khandani B., Nooshadokht M., Shabandoust H., Mohammadi M.A. (2019). Prevalence and molecular subtyping of *Blastocystis* from patients with irritable bowel syndrome, inflammatory bowel disease and chronic urticaria in Iran. Acta Parasitol..

[36] Nourrisson C., Scanzi J., Brunet J., Delbac F., Dapoigny M., Poirier P. (2021). Prokaryotic and eukaryotic fecal microbiota in irritable bowel syndrome patients and healthy individuals colonized with *Blastocystis*. Front. Microbiol..

[37] Stensvold C.R., Alfellani M., Clark C.G. (2012). Levels of genetic diversity vary dramatically between *Blastocystis* subtypes. Infect. Genet. Evol..

[38] Wu Z., Mirza H., Tan K.S.W. (2014). Intra-subtype variation in enteroadhesion accounts for differences in epithelial barrier disruption and is associated with metronidazole resistance in *Blastocystis* subtype-7. PLoS Neglected Trop. Dis..

[39] Alinaghizade A., Mirjalali H., Mohebali M., Stensvold C.R., Rezaeian M. (2017). Inter- and intra-subtype variation of *Blastocystis* subtypes isolated from diarrheic and non-diarrheic patients in Iran. Infect. Genet. Evol..

[40] Gentekaki E., Curtis B.A., Stairs C.W., Klimeš V., Eliáš M., Salas-Leiva D.E., Herman E.K., Eme L., Arias M.C., Henrissat B. (2017). Extreme genome diversity in the hyper-prevalent parasitic eukaryote *Blastocystis*. PLoS Biol..

[41] Billy V., Lhotská Z., Jirků M., Kadlecová O., Frgelecová L., Parfrey L.W., Pomajbíková K.J. (2021). *Blastocystis* colonization alters the gut microbiome and, in some cases, promotes faster recovery from induced colitis. Front. Microbiol..

[42] Scanlan P.D., Stensvold C.R., Rajilić-Stojanović M., Heilig H.G.H.J., De Vos W.M., O’Toole P.W., Cotter P.D. (2014). The microbial eukaryote *Blastocystis* is a prevalent and diverse member of the healthy human gut microbiota. FEMS Microbiol. Ecol..

[43] Kodio A., Coulibaly D., Koné A.K., Konaté S., Doumbo S., Guindo A., Bittar F., Gouriet F., Raoult D., Thera M.A. (2019). *Blastocystis* colonization is associated with increased diversity and altered gut bacterial communities in healthy malian children. Microorganisms.

[44] Even G., Lokmer A., Rodrigues J., Audebert C., Viscogliosi E., Ségurel L., Chabé M. (2021). Changes in the human gut microbiota associated with colonization by *Blastocystis* sp. and *Entamoeba* spp. in non-industrialized populations. Front. Cell. Infect. Microbiol..

[45] Bogoch I.I., Raso G., N’Goran E.K., Marti H.P., Utzinger J. (2006). Differences in microscopic diagnosis of helminths and intestinal protozoa among diagnostic centres. Eur. J. Clin. Microbiol. Infect. Dis..

[46] Stensvold C.R., Arendrup M.C., Jespersgaard C., Molbak K., Nielsen H.V. (2007). Detecting *Blastocystis* using parasitologic and DNA-based methods: A comparative study. Diagn. Microbiol. Infect. Dis..

[47] Coyle C.M., Varughese J., Weiss L.M., Tanowitz H.B. (2012). *Blastocystis*: To treat or not to treat. Clin. Infect. Dis..

[48] Roberts T., Stark D., Harkness J., Ellis J. (2014). Update on the pathogenic potential and treatment options for *Blastocystis* sp.. Gut Pathog..

[49] Roberts T., Ellis J., Harkness J., Marriott D., Stark D. (2014). Treatment failure in patients with chronic *Blastocystis* infection. J. Med. Microbiol..

[50] Subirats M., Borrás R. (2018). *Blastocystis* sp., an emerging parasite with controversial pathogenicity. Should all human cases be treated?. Rev. Clínica Española (Engl. Ed.).

[51] Stojałowska W., Moniuszko A. (1955). Pasożyty przewodu pokarmowego dzieci w żłobkach i przedszkolach Lublina; Parasites of the alimentary tract of children in nurseries and preparatory schools in Lublin. Ann. Univ. Mariae Curie-Skłodowska Lub. -Pol..

[52] Siński E., Bukowska J., Czarnogrecka M., Oralewska B., Świątkowska E., Socha J. (1991). Zarażenie *Cryptosporidium* sp. i *Blastocystis hominis* u dzieci z objawami gastroenterocolitis. Wiadomości Lek..

[53] Raś-Noryńska M., Białkowska J., Sokół R., Piskorz-Ogórek K. (2011). Parasitological stool examination from children without the typical symptoms of parasitic disease. Prz. Epidemiol..

[54] Kowalewska B., Rudzińska M., Zarudzka D., Kotłowski A. (2013). Ocena częstości zarażeń pasożytami jelitowymi wśród pacjentów przychodni Instytutu Medycyny Morskiej i Tropikalnej w Gdyni w okresie ostatnich 30 lat. An evaluation of the intensity of intestinal parasitic infections among patients of out-patient division. Diagn. Lab..

[55] Duda A., Kosik-Bogacka D., Lanocha-Arendarczyk N., Kołodziejczyk L., Lanocha A. (2015). The prevalence of *Blastocystis hominis* and other protozoan parasites in soldiers returning from peacekeeping missions. Am. J. Trop. Med. Hyg..

[56] Lepczyńska M., Dzika E., Stensvold C.R. (2016). Genetic diversity of *Blastocystis* spp. in the human population of the Olsztyn area. Ann. Parasitol..

[57] Korzeniewski K. (2016). Inwazje pasożytami jelitowymi w środowisku dziecięcym Warszawy. Fam. Med. Prim. Care Rev..

[58] Kasprzak J., Szaładzińska B., Smoguła M., Ziuziakowski M. (2017). Intestinal parasites in stool samples and perianal swabs examined by The Voivodeship Sanitary-Epidemiological Station in Bydgoszcz between 2000–2014. Prz. Epidemiol..

[59] Bednarska M., Jankowska I., Pawelas A., Piwczyńska K., Bajer A., Wolska-Kuśnierz B., Wielopolska M., Welc-Falęciak R. (2018). Prevalence of *Cryptosporidium*, *Blastocystis*, and other opportunistic infections in patients with primary and acquired immunodeficiency. Parasitol. Res..

[60] Wesołowska M., Frączkowski M., Rymer W., Janicki P., Poniewierka E., Puszyński G., Kaczmarek A., Sałamatin R. (2019). Molecular subtyping of *Blastocystis* isolates from symptomatic and asymptomatic patients in Lower Silesia, Poland. Ann. Parasitol..

[61] Kaczmarek A., Wesołowska M., Gołąb E., Sałamatin R. (2019). *Blastocystis* spp. infection in young people in Poland. Ann. Parasitol..

[62] Gągała K., Kołodziejczyk L., Kosik-Bogacka D., Pilecka-Rapacz M. (2020). Assessment of the prevalence of intestinal parasite infections among patients at the Laboratory of Department of Biology and Medical Parasitology of the Pomeranian Medical University in Szczecin in the years 1983–2012. Pomeranian J. Life Sci..

[63] Rudzińska M., Kowalewska B., Waleron M., Kalicki M., Sikorska K., Szostakowska B. (2021). Molecular characterization of *Blastocystis* from animals and their caregivers at the Gdańsk zoo (Poland) and the assessment of zoonotic transmission. Biology.

[64] Kosik-Bogacka D., Lepczyńska M., Kot K., Szkup M., Łanocha-Arendarczyk N., Dzika E., Grochans E. (2021). Prevalence, subtypes and risk factors of *Blastocystis* spp. infection among pre- and perimenopausal women. BMC Infect. Dis..

[65] Rudzińska M., Kowalewska B., Kurpas M., Szostakowska B. (2022). Rare Occurrence of *Blastocystis* in pet animals and their owners in the Pomeranian Voivodeship in Poland in the light of literature data. J. Clin. Med..

[66] Kotłowski A. (2012). Blastocystosis—An attempt of evaluation of clinical symptoms and effectivity of metronidazole treatment in different parasitic invasion levels and isolated genotypes of *Blastocystis* sp. among Poles returning from the tropics and not leaving their country. Ann. Acad. Medicae Gedanensis.

[67] Kaczmarek A., Gołąb E., Żarnowska-Prymek H., Rawska A., Jańczak D., Lewicki A., Wesołowska M., Rożej-Bielicka W., Cielecka D., Sałamatin R. (2017). Genetic diversity of *Blastocystis hominis* sensu lato isolated from humans in Poland = Zróżnicowanie genetyczne *Blastocystis hominis* sensu lato wyizolowanych od ludzi w Polsce. Prz. Epidemiol..

[68] Rudzińska M., Kowalewska B., Wąż P., Sikorska K., Szostakowska B. (2019). *Blastocystis* subtypes isolated from travelers and non-travelers from the north of Poland—A single center study. Infect. Genet. Evol..

[69] Kaczmarek A., Rocka A., Wesołowska M., Gołąb E., Sałamatin R. (2020). *Blastocystis* isolates from a dog and their owners presenting with chronic diarrhoea. Dogs as reservoirs of *Blastocystis*: Research in Poland and worldwide. Ann. Parasitol..

[70] Lewicki A., Rożej-Bielicka W., Sałamatin R. (2016). *Blastocystis hominis* s. l ST6-parasite of chickens-new zoonotic agent in Poland. Ann. Parasitol..

[71] Kaczmarek A., Lewicki A., Dziedzic K., Sulecki K., Rożej-Bielicka W., Wesołowska M., Gołąb E. (2019). A survey of *Blastocystis* in domestic chickens from Poland and Madagascar. Ann. Parasitol..

[72] Wesołowska M., Paszta W., Michrowska A., Piekarska J., Wesołowska M., Gorczykowski M., Kaczmarek A., Sałamatin R. (2019). Molecular characterization of *Blastocystis* subtypes isolated from various mammalian groups living in Wrocław ZOO, Poland. Ann. Parasitol..

[73] Rudzińska M., Kowalewska B., Szostakowska B., Grzybek M., Sikorska K., Świątalska A. (2020). First report on the occurrence and subtypes of *Blastocystis* in pigs in Poland using sequence-tagged-site pcr and barcode region sequencing. Pathogens.

[74] Falkowski P., Gaweł A., Bobrek K. (2022). Prevalence of *Blastocystis* in geese reproductive flocks. Animals.

[75] Kaczmarek A., Sobociński W., Wesołowska M., Gołąb E., Kołodziej-Sobocińska M., Sałamatin R. (2021). *Blastocystis* occurrence and subtype diversity in wild European terrestrial mammals—The case of Białowieża Primeval Forest (NE Poland). Int. J. Parasitol. Parasites Wildl..

[76] Sinniah B., Hassan A.K.R., Sabaridah I., Soe M.M., Ibrahim Z., Ali O. (2014). Prevalence of intestinal parasitic infections among communities living in different habitats and its comparison with one hundred and one studies conducted over the past 42 years (1970 to 2013) in Malaysia. Trop. Biomed..

[77] El Safadi D., Cian A., Nourrisson C., Pereira B., Morelle C., Bastien P., Bellanger A.P., Botterel F., Candolfi E., Desoubeaux G. (2016). Prevalence, risk factors for infection and subtype distribution of the intestinal parasite *Blastocystis* sp. from a large-scale multi-center study in France. BMC Infect. Dis..

[78] Barbosa C.V., Barreto M.M., de Jesus Andrade R., Sodré F., D’Avila-Levy C.M., Peralta J.M., Igreja R.P., de Macedo H.W., Santos H.L.C. (2018). Intestinal parasite infections in a rural community of Rio de Janeiro (Brazil): Prevalence and genetic diversity of *Blastocystis* subtypes. PLoS ONE.

[79] Javanmard E., Niyyati M., Ghasemi E., Mirjalali H., Asadzadeh Aghdaei H., Zali M.R. (2018). Impacts of human development index and climate conditions on prevalence of *Blastocystis*: A systematic review and meta-analysis. Acta Trop..

[80] Mondot S., Poirier P., Abou-Bacar A., Greigert V., Brunet J., Nourrisson C., Randrianarivelojosia M., Razafindrakoto J.L., Morel E., Rakotomalala R.S. (2021). Parasites and diet as main drivers of the Malagasy gut microbiome richness and function. Sci. Rep..

[81] Alfellani M.A., Stensvold C.R., Vidal-Lapiedra A., Onuoha E.S.U., Fagbenro-Beyioku A.F., Clark C.G. (2013). Variable geographic distribution of *Blastocystis* subtypes and its potential implications. Acta Trop..

[82] Popruk S., Adao D.E.V., Rivera W.L. (2021). Epidemiology and subtype distribution of *Blastocystis* in humans: A review. Infect. Genet. Evol..

[83] van Hattem J.M., Arcilla M.S., Grobusch M.P., Bart A., Bootsma M.C., van Genderen P.J., van Gool T., Goorhuis A., van Hellemond J.J., Molenkamp R. (2017). Travel-related acquisition of diarrhoeagenic bacteria, enteral viruses and parasites in a prospective cohort of 98 Dutch travellers. Travel Med. Infect. Dis..

[84] Nowak P., Jochymek M. (2007). Występowanie pasożytów jelitowych człowieka w wybranych populacjach na terenie Krakowa w latach 2000–2006 na podstawie badań parazytologicznych kału przeprowadzonych w Laboratorium Parazytologii Wojewódzkiej Stacji Sanitarno−Epidemiologicznej. Wiadomości Parazytol..

[85] Saksirisampant W., Prownebon J., Kulkumthorn M., Yenthakam S., Janpla S., Nuchprayoon S. (2006). Prevalence of intestinal parasitic infections among school children in the central region of Thailand. J. Med. Assoc. Thail. = Chotmaihet Thangphaet.

[86] Saksirisampant W., Nuchprayoon S., Wiwanitkit V., Yenthakam S., Ampavasiri A. (2003). Intestinal parasitic infestations among children in an orphanage in Pathum Thani province. J. Med. Assoc. Thail. = Chotmaihet Thangphaet.

[87] Mohammad N.A., Al-Mekhlafi H.M., Anuar T.S. (2018). Subtype distribution of *Blastocystis* isolated from humans and associated animals in an indigenous community with poor hygiene in Peninsular Malaysia. Trop. Biomed..

[88] Mohammad N.A., Al-Mekhlafi H.M., Moktar N., Anuar T.S. (2017). Prevalence and risk factors of *Blastocystis* infection among underprivileged communities in rural Malaysia. Asian Pac. J. Trop. Med..

[89] Hidalgo L., Salvador F., Sulleiro E., López I., Balladares M., García E., Paz C., Sánchez-Montalvá A., Bosch-Nicolau P., Sao-Avilés A. (2019). Evaluation of risk factors associated to detection of *Blastocystis* sp. in fecal samples in population from Barcelona, Spain: A case-control study. Eur. J. Clin. Microbiol. Infect. Dis..

[90] Roberts T., Stark D., Harkness J., Ellis J. (2013). Subtype distribution of *Blastocystis* isolates identified in a Sydney population and pathogenic potential of *Blastocystis*. Eur. J. Clin. Microbiol. Infect. Dis..

[91] Jantermtor S., Pinlaor P., Sawadpanich K., Pinlaor S., Sangka A., Wilailuckana C., Wongsena W., Yoshikawa H. (2013). Subtype identification of *Blastocystis* spp. isolated from patients in a major hospital in north-eastern Thailand. Parasitol. Res..

[92] Scanlan P.D., Knight R., Song S.J., Ackermann G., Cotter P.D. (2016). Prevalence and genetic diversity of *Blastocystis* in family units living in the United States. Infect. Genet. Evol..

[93] Piubelli C., Soleymanpoor H., Giorli G., Formenti F., Buonfrate D., Bisoffi Z., Perandin F. (2019). *Blastocystis* prevalence and subtypes in autochthonous and immigrant patients in a referral centre for parasitic infections in Italy. PLoS ONE.

[94] Seyer A., Karasartova D., Ruh E., Gureser A.S., Turgal E., Imir T., Taylan-Ozkan A. (2017). Epidemiology and prevalence of *Blastocystis* spp. In North Cyprus. Am. J. Trop. Med. Hyg..

[95] Tito R.Y., Chaffron S., Caenepeel C., Lima-Mendez G., Wang J., Vieira-Silva S., Falony G., Hildebrand F., Darzi Y., Rymenans L. (2019). Population-level analysis of *Blastocystis* subtype prevalence and variation in the human gut microbiota. Gut.

[96] Lhotska Z., Jirku M., Hlożkova O., Brożova K., Jirsova D., Stensvold C.R., Kolisko M., Pomajbikowa K. (2020). A Study on the prevalence and subtype diversity of the intestinal protist *Blastocystis* sp. in a gut-healthy human population in the Czech Republic. Front. Cell. Infect. Microbiol..

[97] Roberts T., Barratt J., Harkness J., Ellis J., Stark D. (2011). Comparison of microscopy, culture, and conventional polymerase chain reaction for detection of *Blastocystis* sp. In clinical stool samples. Am. J. Trop. Med. Hyg..

[98] Sagnuankiat S., Wanichsuwan M., Bhunnachet E., Jungarat N., Panraksa K., Komalamisra C., Maipanich W., Yoonuan T., Pubampen S., Adisakwattana P. (2016). Health status of immigrant children and environmental survey of child daycare centers in Samut Sakhon Province, Thailand. J. Immigr. Minor. Health.

[99] Dudlová A., Jarčuška P., Jurišová S., Vasilková Z., Krčméry V., Juriš P. (2018). Prevalence of non-pathogenic types of gastrointestinal protozoa in population in Slovakia and their potential importance in the aspect of public health. Acta Parasitol..

[100] Légeret C., Rüttimann C., Fankhauser H., Köhler H. (2021). Parasitic infections in Swiss children: Are we overtesting?. BMC Gastroenterol..

[101] Nithyamathi K., Chandramathi S., Kumar S. (2016). Predominance of *Blastocystis* sp. infection among school children in Peninsular. PLoS ONE.

[102] Chandrasena T., Alwis D., de Silva L., Morel R., de Silva N. (2004). Intestinal parasitosis and the nutritional status of Veddah children in Sri Lanka. Southeast Asian J. Trop. Med. Public Health.

[103] Cinek O., Polackova K., Odeh R., Alassaf A., Kramná L., Ibekwe M.A.U., Majaliwa E.S., Ahmadov G., Elmahi B.M.E., Mekki H. (2021). *Blastocystis* in the faeces of children from six distant countries: Prevalence, quantity, subtypes and the relation to the gut bacteriome. Parasites Vectors.

[104] Villamizar X., Higuera A., Herrera G., Vasquez-A L.R., Buitron L., Muñoz L.M., Gonzalez-C F.E., Lopez M.C., Giraldo J.C., Ramírez J.D. (2019). Molecular and descriptive epidemiology of intestinal protozoan parasites of children and their pets in Cauca, Colombia: A cross-sectional study. BMC Infect. Dis..

[105] Dacal E., Saugar J.M., De Lucio A., Hernández-De-Mingo M., Robinson E., Köster P.C., Aznar-Ruiz-De-Alegría M.L., Espasa M., Ninda A., Gandasegui J. (2018). Prevalence and molecular characterization *of Strongyloides stercoralis, Giardia duodenalis, Cryptosporidium* spp., and *Blastocystis* spp. isolates in school children in Cubal, Western Angola. Parasites Vectors.

[106] Amato Neto V., Rodríguez Alarcon R.S., Gakiya E., Ferreira C.S., Bezerra R.C., dos Santos A.G. (2004). Elevada porcentagem de blastocistose em escolares de São Paulo, SP. Rev. Da Soc. Bras. De Med. Trop..

[107] Oliveira-Arbex A.P., David É.B., Guimarães S. (2018). *Blastocystis* genetic diversity among children of low-income daycare center in Southeastern Brazil. Infect. Genet. Evol..

[108] Rebolla M.F., Silva E.M., Gomes J.F., Falcão A.X., Rebolla M.V.F., Franco R.M.B. (2016). High prevalence of *Blastocystis* spp. infection in children and staff members attending public urban schools in São Paulo State, Brazil. Rev. Do Inst. De Med. Trop. De Sao Paulo.

[109] Dogan N., Aydin M., Tuzemen N.U., Dinleyici E.C., Oguz I., Dogruman-Al F. (2017). Subtype distribution of *Blastocystis* spp. isolated from children in Eskisehir, Turkey. Parasitol. Int..

[110] Baldo E.T., Belizario V.Y., De Leon W.U., Kong H.H., Chung D. (2004). Il Infection status of intestinal parasites in children living in residential institutions in Metro Manila, the Philippines. Korean J. Parasitol..

[111] Poulsen C.S., Efunshile A.M., Nelson J.A., Stensvold C.R. (2016). Epidemiological aspects of *Blastocystis* colonization in children in Ilero, Nigeria. Am. J. Trop. Med. Hyg..

[112] El Safadi D., Gaayeb L., Meloni D., Cian A., Poirier P., Wawrzyniak I., Delbac F., Dabboussi F., Delhaes L., Seck M. (2014). Children of Senegal River Basin show the highest prevalence of *Blastocystis* sp. ever observed worldwide. BMC Infect. Dis..

[113] Ahmed S.A., El-Mahallawy H.S., Mohamed S.F., Angelici M.C., Hasapis K., Saber T., Karanis P. (2022). Subtypes and phylogenetic analysis of *Blastocystis* sp. Isolates from West Ismailia, Egypt. Sci. Rep..

[114] Rivera W.L., Tan M.A.V. (2005). Molecular characterization of *Blastocystis* isolates in the Philippines by riboprinting. Parasitol. Res..

[115] Motazedian H., Ghasemi H., Sadjjadi S.M. (2008). Genomic diversity of *Blastocystis hominis* from patients in southern Iran. Ann. Trop. Med. Parasitol..

[116] Yakoob J., Jafri W., Beg M.A., Abbas Z., Naz S., Islam M., Khan R. (2010). Irritable bowel syndrome: Is it associated with genotypes of *Blastocystis hominis*. Parasitol. Res..

[117] Poirier P., Wawrzyniak I., Albert A., El Alaoui H., Delbac F., Livrelli V. (2011). Development and evaluation of a real-time PCR assay for detection and quantification of *Blastocystis* parasites in human stool samples: Prospective study of patients with hematological malignancies. J. Clin. Microbiol..

[118] Tan K.S.W., Mirza H., Teo J.D.W., Wu B., MacAry P.A. (2010). Current views on the clinical relevance of *Blastocystis* spp.. Curr. Infect. Dis. Rep..

[119] Jiménez P., Muñoz M., Ramírez J.D. (2022). An update on the distribution of *Blastocystis* subtypes in the Americas. Heliyon.

[120] Bart A., Wentink-Bonnema E.M., Gilis H., Verhaar N., Wassenaar C.J., van Vugt M., Goorhuis A., van Gool T. (2013). Diagnosis and subtype analysis of *Blastocystis* sp.in 442 patients in a hospital setting in the Netherlands. BMC Infect. Dis..

[121] Engsbro A.L., Stensvold C.R., Nielsen H.V., Bytzer P. (2014). Prevalence, incidence, and risk factors of intestinal parasites in Danish primary care patients with irritable bowel syndrome. Scand. J. Infect. Dis..

[122] Sheikh S., Asghari A., Sadraei J., Pirestani M., Zare M. (2020). Blastocystis sp. Subtype 9: As the first reported subtype in patients with schizophrenia in Iran. SN Compr. Clin. Med..

[123] Yoshikawa H., Iwamasa A. (2016). Human *Blastocystis* subtyping with subtype-specific primers developed from unique sequences of the SSU rRNA gene. Parasitol. Int..

[124] Scicluna S.M., Tawari B., Clark C.G. (2006). DNA barcoding of *Blastocystis*. Protist.

[125] Scanlan P.D., Stensvold C.R., Cotter P.D. (2015). Development and application of a *Blastocystis* subtype-specific PCR assay reveals that mixed-subtype infections are common in a healthy human population. Appl. Environ. Microbiol..

[126] Yoshikawa H., Tokoro M., Nagamoto T., Arayama S., Asih P.B.S., Rozi I.E., Syafruddin D. (2016). Molecular survey of *Blastocystis* sp. from humans and associated animals in an Indonesian community with poor hygiene. Parasitol. Int..

[127] Belleza M.L.B., Reyes J.C.B., Tongol-Rivera P.N., Rivera W.L. (2016). Subtype analysis of *Blastocystis* sp. isolates from human and canine hosts in an urban community in the Philippines. Parasitol. Int..

[128] Cian A., El Safadi D., Osman M., Moriniere R., Gantois N., Benamrouz-Vanneste S., Delgado-Viscogliosi P., Guyot K., Li L.L., Monchy S. (2017). Molecular epidemiology of *Blastocystis* sp. in various animal groups from two French zoos and evaluation of potential zoonotic risk. PLoS ONE.

[129] Cai J., Qiao J., Zhang X., Jin X., Ren J., Ma Q., XM W. (2009). Observation on intestinal parasitic morphology and infection in captive rare wildlife in Shaanxi Province. Chin. J. Zool..

[130] Alfellani M.A., Taner-Mulla D., Jacob A.S., Imeede C.A., Yoshikawa H., Stensvold C.R., Clark C.G. (2013). Genetic Diversity of *Blastocystis* in Livestock and Zoo Animals. Protist.

[131] Tan T.C., Tan P., Sharma R., Sugnaseelan S., Suresh K. (2013). Genetic diversity of caprine *Blastocystis* from Peninsular Malaysia. Parasitol. Res..

[132] Betts E.L., Gentekaki E., Thomasz A., Breakell V., Carpenter A.I., Tsaousis A.D. (2017). Genetic diversity of *Blastocystis* in non- primate animals. Parasitology.

[133] Song J.K., Hu R.S., Fan X.C., Wang S.S., Zhang H.J., Zhao G.H. (2017). Molecular characterization of *Blastocystis* from pigs in Shaanxi province of China. Acta Trop..

[134] Valença-Barbosa C., Do Bomfim T.C.B., Teixeira B.R., Gentile R., Da Costa Neto S.F., Magalhães B.S.N., De Almeida Balthazar D., Da Silva F.A., Biot R., D’Avila Levy C.M. (2019). Molecular epidemiology of *Blastocystis* isolated from animals in the state of Rio de Janeiro, Brazil. PLoS ONE.

[135] Lee H., Seo M.-G., Oem J.-K., Kim Y.-S., Lee S.-Y., Kim J., Jeong H., Jheong W.-H., Kim Y., Lee W.-J. (2020). Molecular detection and subtyping of *Blastocystis* detected in wild boars (*Sus scrofa*) in South Korea. J. Wildl. Dis..

[136] Wang J., Gong B., Yang F., Zhang W., Zheng Y., Liu A. (2018). Subtype distribution and genetic characterizations of *Blastocystis* in pigs, cattle, sheep and goats in north-eastern China’s Heilongjiang Province. Infect. Genet. Evol..

[137] Denoeud F., Roussel M., Noel B., Wawrzyniak I., Da Silva C., Diogon M., Viscogliosi E., Brochier-Armanet C., Couloux A., Poulain J. (2011). Genome sequence of the stramenopile *Blastocystis*, a human anaerobic parasite. Genome Biol..

[138] AbuOdeh R., Ezzedine S., Madkour M., Stensvold C.R., Samie A., Nasrallah G., AlAbsi E., ElBakri A. (2019). Molecular subtyping of *Blastocystis* from diverse animals in the United Arab Emirates. Protist.

[139] Adamska M. (2022). First report of *Blastocystis* sp. subtypes in natural water bodies in north-western Poland: A one-year monitoring. Int. J. Environ. Health Res..

[140] Słodkowicz-Kowalska A., Majewska A.C., Rzymski P., Skrzypczak Ł., Werner A. (2015). Human waterborne protozoan parasites in freshwater bivalves (*Anodonta anatina* and *Unio tumidus*) as potential indicators of fecal pollution in urban reservoir. Limnologica.

[141] Leelayoova S., Siripattanapipong S., Thathaisong U., Naaglor T., Taamasri P., Piyaraj P., Mungthin M. (2008). Drinking water: A possible source of *Blastocystis* spp. subtype 1 infection in schoolchildren of a rural community in central Thailand. Am. J. Trop. Med. Hyg..

[142] Ithoi I., Jali A., Mak J.W., Yusoff W., Sulaiman W., Mahmud R. (2011). Occurrence of *Blastocystis* in water of two rivers from recreational areas in Malaysia. J. Parasitol. Res..

[143] Khalifa R.M.A., Ahmad A.K., Abdel-Hafeez E.H., Mosllem F.A. (2014). Present status of protozoan pathogens causing waterborne disease in northern part of El-Minia Governorate, Egypt. J. Egypt. Soc. Parasitol..

[144] Noradilah S.A., Lee I.L., Anuar T.S., Salleh F.M., Manap S.N.A.A., Mohtar N.S.H.M., Azrul S.M., Abdullah W.O., Moktar N. (2016). Occurrence of *Blastocystis* sp. in water catchments at Malay villages and Aboriginal settlement during wet and dry seasons in Peninsular Malaysia. PeerJ.

[145] Koloren Z., Gulabi B.B., Karanis P. (2018). Molecular identification of *Blastocystis* sp. subtypes in water samples collected from Black Sea, Turkey. Acta Trop..

[146] Leelayoova S., Siripattanapipong S., Naaglor T., Taamasri P., Mungthin M. (2009). Prevalence of intestinal parasitic infections in military personnel and military dogs, Thailand. J. Med. Assoc. Thail. = Chotmaihet Thangphaet.

[147] Lee L.I., Chye T.T., Karmacharya B.M., Govind S.K. (2012). *Blastocystis* sp.: Waterborne zoonotic organism, a possibility?. Parasites Vectors.

[148] Suresh K., Smith H., Tan T. (2005). Viable *Blastocystis* cysts in Scottish and Malaysian sewage samples. Appl. Environ. Microbiol..

[149] Banaticla J.E.G., Rivera W.L. (2011). Detection and subtype identification of *Blastocystis* isolates from wastewater samples in the Philippines. J. Water Health.

[150] Pérez-Cordón G., Rosales M.J., Del Mar Gavira M., Valdez R.A., Vargas F., Córdova O. (2007). Finding of *Blastocystis* sp. in bivalves of the genus *Donax*. Rev. Peru. De Biol..

[151] Eme L., Gentekaki L., Curtis B., Archibald J.M., Roger A.J. (2017). Lateral gene transfer in the adaptation of the anaerobic parasite *Blastocystis* to the gut. Curr. Biol..

